# Effects of a Five-Year Citywide Intervention Program To Control *Aedes aegypti* and Prevent Dengue Outbreaks in Northern Argentina

**DOI:** 10.1371/journal.pntd.0000427

**Published:** 2009-04-28

**Authors:** Ricardo E. Gürtler, Fernando M. Garelli, Héctor D. Coto

**Affiliations:** 1 Laboratorio de Eco-Epidemiología, Facultad de Ciencias Exactas y Naturales, Universidad de Buenos Aires, Buenos Aires, Argentina; 2 Fundación Mundo Sano, Buenos Aires, Argentina; Universidade de São Paulo, Brazil

## Abstract

**Background:**

Dengue has propagated widely through the Americas. Most countries have not been able to maintain permanent larval mosquito control programs, and the long-term effects of control actions have rarely been documented.

**Methodology:**

The study design was based on a before-and-after citywide assessment of *Aedes aegypti* larval indices and the reported incidence of dengue in Clorinda, northeastern Argentina, over 2003–2007. Interventions were mainly based on focal treatment with larvicides of every mosquito developmental site every four months (14 cycles), combined with limited source reduction efforts and ultra-low-volume insecticide spraying during emergency operations. The program conducted 120,000 house searches for mosquito developmental sites and 37,000 larvicide applications.

**Principal Findings:**

Random-effects regression models showed that Breteau indices declined significantly in nearly all focal cycles compared to pre-intervention indices clustered by neighborhood, after allowing for lagged effects of temperature and rainfall, baseline Breteau index, and surveillance coverage. Significant heterogeneity between neighborhoods was revealed. Larval indices seldom fell to 0 shortly after interventions at the same blocks. Large water-storage containers were the most abundant and likely to be infested. The reported incidence of dengue cases declined from 10.4 per 10,000 in 2000 (by DEN-1) to 0 from 2001 to 2006, and then rose to 4.5 cases per 10,000 in 2007 (by DEN-3). In neighboring Paraguay, the reported incidence of dengue in 2007 was 30.6 times higher than that in Clorinda.

**Conclusions:**

Control interventions exerted significant impacts on larval indices but failed to keep them below target levels during every summer, achieved sustained community acceptance, most likely prevented new dengue outbreaks over 2003–2006, and limited to a large degree the 2007 outbreak. For further improvement, a shift is needed towards a multifaceted program with intensified coverage and source reduction efforts, lids or insecticide-treated covers to water-storage containers, and a broad social participation aiming at long-term sustainability.

## Author Summary

Dengue has become the most important viral disease of humans transmitted by arthropods in tropical and subtropical urban regions. Most countries have not been able to maintain permanent larval mosquito control programs against *Aedes aegypti*, the main vector of dengue and yellow fever, partly because of misguided reliance on using insecticidal space sprays to kill adult mosquitoes. Control actions targeting larvae are based on regular treatment with insecticides of every mosquito developmental site, usually artificial containers found inside or around human dwellings. The reasons why sustained larval control programs cannot reduce infestations to the desired levels have rarely been investigated. A five-year intervention program conducted in Clorinda, northeastern Argentina, reduced significantly larval infestations compared to pre-intervention levels. Infestations depended on weather variations, pre-intervention larval infestation, and the percentage of houses that were visited and treated with larvicides. Although the program did not reach the low levels of infestation desired, it most likely prevented or limited new local dengue outbreaks. Large containers for permanent water storage were the most important mosquito development site. For further improvements, a multifaceted intervention program is needed. It should include intensified surveillance and treatment coverage with larvicides that last longer, more efforts on reducing the potential number of mosquito development sites, and a broad social participation aiming at long-term sustainability.

## Introduction

The global incidence of dengue has increased exponentially since 1955 to reach 1–50 million infections per year in 2000–2005 [1]. Dengue has become the most important arboviral disease of humans, and an increasing urban health and economic problem in tropical and subtropical regions worldwide. Classic dengue fever (DF) and its more severe forms, dengue hemorrhagic fever (DHF) and dengue shock syndrome (DSS), have expanded worldwide from Southeast Asia since 1946 [2]. The four existing serotypes of dengue virus (DEN-1, DEN-2, DEN-3 and DEN-4) only confer life-long immunity against reinfection by the same serotype, and subsequent infections with a different serotype enhance the likelihood of DHF/DSS [3],[4]. There are currently no anti-dengue drugs available, and promising vaccines still await efficacy trials [5]. *Aedes aegypti*, a domestic mosquito species that develops in water-holding containers, is the main vector of dengue and urban yellow fever. Current dengue control strategies seek to reduce *Ae. aegypti* abundance; optimize diagnosis and treatment of dengue cases, and decrease the frequency, magnitude and severity of dengue epidemics through integrated control strategies [6].

Dengue became a recognized public health threat in the western hemisphere after the 1981 Cuban epidemic, the first major DHF/DSS outbreak in the region [7]. Eradication programs initiated in 1915 achieved the apparent elimination of *Ae. aegypti* from most of the region by 1970 utilizing vertically-structured programs that included full-coverage source reduction supplemented with perifocal insecticide spraying with DDT [7],[8]. During the 1970s, however, *Ae. aegypti* reinfested most of the countries from where it had been eliminated and is no longer considered a target for elimination [7]. Despite the transition from eradication to control, very little information on the long-term effects of vector control actions are available [9],[10] and success stories are limited. Moreover, “Experience of vector control programs at various program levels and of successful and unsuccessful disease and vector surveillance systems needs to be recorded to allow adoption of best practices in other places” [1].

After a contained dengue outbreak by DEN-1 and DEN-4 in Roraima (northern Brazil) in 1981–1982, the Southern Cone countries of South America began to suffer major DF outbreaks in 1986 (Brazil), 1987–1988 (Bolivia), 1988–1989 (Paraguay), 1998 (Argentina) and 2002 (the island of Pascua, Chile) [7],[11]. Uruguay and continental Chile have remained free of local dengue outbreaks, though they notified imported cases in 2006–2007. Northern Peru has had dengue activity since 1990, but the southern departments have remained free of *Ae. aegypti* and dengue so far. In Brazil, the number of reported dengue cases between 1995 and 2007 ranged from approximately 100,000 to 800,000 per year [11]–[14]. The northeastern and southeastern Brazilian states were invaded by DEN-1 in 1986–1987; by DEN-2 in 1990–1991, and by DEN-3 in December 2000. DHF/DSS cases have occurred every year in Brazil since 1994 [14] and in Bolivia since 2002.

In Argentina, the last epidemic outbreak of DF before the eradication era occurred in eastern and northeastern provinces in 1926 [15]. Declared eradicated in 1963, the presence of *Ae. aegypti* was again detected in two northeastearn provinces in 1986, and most provinces north to 35°S were found to be reinfested by 1999 [16]. The number of localities infested currently exceeds those recorded before the eradication era [17]. The first local outbreak of DF (by DEN-2) occurred in the northwestern Province of Salta in 1998 [18],[19], and was most likely linked to an underreported outbreak of DEN-2 emerging in Bolivia in 1996 and 1997 [20],[21]. The second outbreak of dengue (by DEN-1) occurred in the northeastern provinces of Misiones and Formosa in 2000, on the border with Paraguay where a massive DEN-1 outbreak occurred in 1999–2000 [22]. The emergence of DEN-3 in Salta in early 2003 (co-circulating with DEN-1 and DEN-2) was shortly followed in 2004 by significant outbreaks of DEN-3 there and in the neighboring provinces of Jujuy and western Formosa [23], and in 2006 in Salta and Misiones (by DEN-2 and DEN-3). The historical pattern of dengue case notifications in the Southern Cone between 1995 and 2007 shows increasing hyperendemic transmission of several serotypes with multiannual fluctuations in Brazil, Bolivia and Paraguay, with epidemic behavior in northern Argentina.

Clorinda, the most affected city in Formosa during the 2000 outbreak, is located in one of the high-risk zones of potential dengue transmission in Argentina [24]. In collaboration with the local Municipality and other health and research institutions, Fundación Mundo Sano (FMS) launched a citywide control program in late 2002 with the objective of “reducing the risk of occurrence of autochthonous cases of dengue in Clorinda and its area of influence through the application of strategies that reduce the population abundance of *Ae. aegypti*”. The intervention program had a health promotion and education component but relied primarily on the systematic application of larvicides combined with source reduction efforts. Between 2003 and 2007, the program conducted and monitored 14 cycles of focal treatment, with only the first previously reported [25]. As part of a cooperative agreement between FMS and the University of Buenos Aires established in late 2006, we herein describe the implemented intervention program and assess the long-term effects of vector suppressive actions on *Ae. aegypti* larval indices and on the reported incidence of dengue during the five-year period. We also sought to diagnose major limitations in the control strategy conducted, and prescribe possible solutions for improved vector control.

## Methods

### Study site

The city of Clorinda (25°17′S, 57°43′W, Formosa, Argentina) is located on the southern margin of Pilcomayo River at 45 km from the city of Asunción (population, 519,000 people in 2005) in Paraguay (Figure S1). Clorinda had 38 identified neighborhoods with 10,752 houses and 47,240 inhabitants in 2001, and approximately 16,000 houses and 49,000 people in late 2007; individual neighborhood size differed greatly from <10 to 133 blocks [26]. Nearly 25% of the population had unsatisfied basic needs (i.e., an index of poverty that combines lack of adequate housing and of tap water, crowding, and low income), and 15% of the houses were of mud-and-thatch construction with earthen floors. Approximately 2,000–7,000 people were estimated to commute daily between Clorinda and Paraguay through two bridges.

Although the water service covers most of the city, the distribution of potable water has traditionally been rather discontinuous and unreliable. This fact determines that many residents store water in tanks, barrels and drums or construct wells. The Municipality supplies potable water in community tanks in some peripheral neighborhoods, but this does not fully satisfy the demand. Solid garbage disposal is discontinuous, inappropriate and mainly covers the most affluent neighborhoods in downtown Clorinda.

### Weather patterns

**Figure 1:**
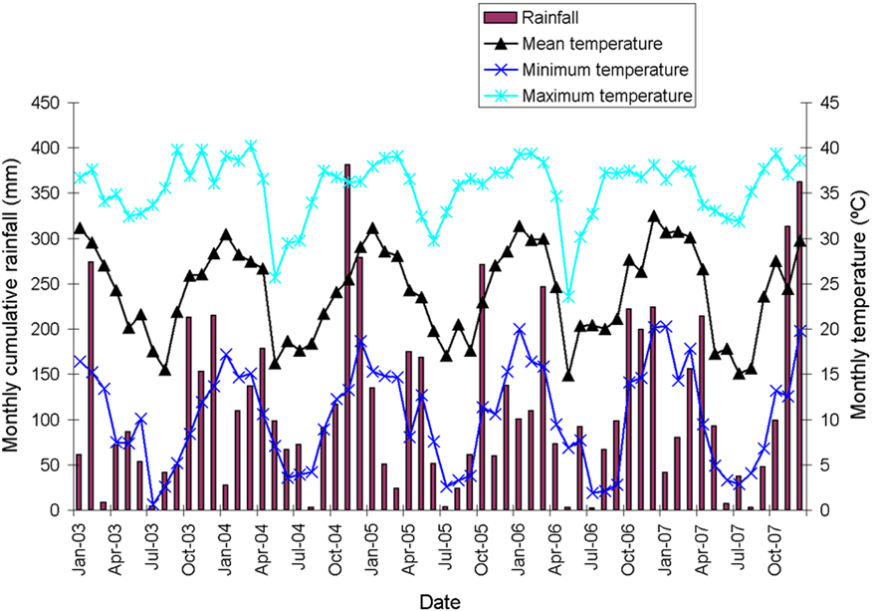
Distribution of monthly cumulative rainfall and monthly mean temperatures over the intervention period in Clorinda, Argentina, January 2003–December 2007.

Meteorological data were collected from January 1, 2003 to December 31, 2007 by a local weather station run by Cooperativa de Provisión de Obras y Servicios Públicos Clorinda Limitada. Mean temperature was 23.1°C, with mean monthly maxima (32.6–34.8°C) in January–February and minima (8.9–12.3°C) in May–August (Figure 1). Annual cumulative rainfall ranged from 1,160 to 1,559 mm/year of which 997–1,160 mm occurred between October and April. Mean relative humidity was 75% (mean monthly maximum, 93%, and minimum, 52%).

### Background data

Dengue is a reportable disease in Argentina. Prior to 2000, dengue virus activity was limited in Clorinda (i.e., 36 imported cases in 1999). In February–May 2000 Clorinda experienced a classic-dengue outbreak with 529 suspected and 74 serologically confirmed cases [19],[27]. Most cases were 15–49 years of age, and one-third of them reported travel history to Paraguay before the onset of symptoms [27]. In immediate response to this outbreak, febrile syndromic surveillance commenced at the public hospitals and health care centers, and emergency vector control operations (focal treatment with 1% temephos, indoor space spraying with pyrethroid insecticides, citywide vehicle-mounted ULV insecticide spraying, and elimination of discarded containers in the least affluent neighborhoods) were initiated. Vector suppressive actions only covered 5% of Clorinda during the second semester of 2001, when the overall prevalence of IgG antibodies to dengue virus was 11.9% (Elena Pedroni, unpublished data, December 2001). Estimates of house and Breteau indices were 22% and 54, respectively. Emergency vector control operations over three weeks reduced house index only by ∼45% shortly after interventions.

### Study design

In collaboration with the Municipality of Clorinda, the Ministry of Health of Argentina and researchers from Centro de Investigaciones de Plagas e Insecticidas (CIPEIN), FMS launched a citywide control program in late 2002 following general guidelines for dengue control programs [7]. Originated in the yellow fever tradition, the desired control targets were a house index <1% and a Breteau index <5. The preparation phase started in November 2002 with a media campaign in radio, television and newspapers, supplemented by the distribution of 5,000 posters and 20,000 leaflets to inform the community and to request that access to houses was given to vector control personnel [25]. Field activities were carried out by 40 community members affiliated to a welfare support plan run by the Municipality of Clorinda. This task force had previously been trained by vector control personnel and researchers on the basics of dengue and *Ae. aegypti* biology, geographic reconnaissance, collection and identification of immature stages of *Ae. aegypti*, application of larvicides, treatment and disposal of discarded containers, how to interview householders and to record data in household visit forms.

#### Preliminary survey

To establish preliminary infestation levels in domestic and peridomestic habitats and collect information to guide subsequent control operations, a random house survey of 1,808 occupied houses was conducted between November 18 and December 13, 2002. Two-person teams inspected each house for water-filled containers infested with larvae or pupae of *Ae. aegypti*. Larval samples from each positive container were collected using ladles and pipettes, placed in test tubes, identified and recorded in household visit forms.

#### Focal cycles

A total of 14 cycles of focal treatment aiming at complete coverage was conducted at almost four-month intervals (mean, 115 days; standard deviation, SD, 29 days) from March 2003 to December 2007 (Table 1). An average of 33 (SD, 7) people participated in each cycle. The total local labor effort over the five-year period was 1,820 person-months. In addition to the container inspections described in the preliminary surveys, field teams also (1) removed or emptied disposable containers (manual treatment); (2) encouraged householders to manage containers on their property, and (3) treated water-storage containers (non-drinking water) with appropriate 1% temephos in sand granules at 1 mg per liter, or less frequently, with *Bacillus thuringiensis israelensis* (BTI, VectobacR, Bayer) applied as rings of prolonged residual power. Field teams recorded the number of residents and grams of larvicide applied in each house, but did not record the number of containers treated manually. Inspection and larviciding efforts in cemeteries and used tire lots were conducted during each focal cycle. Coverage was mapped and continuously updated so that multiple attempts could be made to gain access to as many homes as possible. Occupied houses that could not be inspected on the first visit were re-visited at the end of each focal treatment cycle to enhance surveillance coverage. At the community level, educational efforts were supplemented by nine community workshops conducted in schools distributed throughout the city (one cycle only at the first cycle).

**Table 1:** Summary of interventions and larval indices at the preliminary survey and over focal treatment cycles 1–14 in Clorinda, Argentina, 2002–2007.

Focal cycle	Date of beginning	Duration (days)	MPI	Number of houses					House index	Breteau index	Number of houses sprayed with ULV
				Inspected	Treated	Closed	Refusing access	Visited			
Preliminary	18/11/2002	25	−3	1808	0	NA	NA	1808	19.5	22.5	0
1	03/10/2003	141	0	7512	3170	2621	724	10857	13.7	19.0	1195
2	08/01/2003	131	5	7822	2842	3213	553	11588	3.7	4.8	623
3	01/05/2004	97	10	7430	2221	2560	328	10318	7.3	9.2	750
4	14/04/2004	79	13	7168	2250	2657	251	10076	8.6	10.6	0
5	07/05/2004	133	16	7021	2040	3013	286	10320	3.3	3.9	227
6	19/11/2004	87	21	7137	2123	2586	289	10012	6.5	8.3	1552
7	17/02/2005	71	24	7414	2314	2674	351	10439	5.2	5.5	688
8	14/06/2005	124	29	8219	2688	3208	566	11993	5.2	6.8	0
9	26/10/2005	128	33	7845	2183	3244	533	11622	4.6	5.8	0
10	16/02/2006	56	36	5587	1833	1644	261	7492	11.7	16.6	426*
11	15/05/2006	99	38	9832	2953	3854	494	14180	6.3	7.9	0
12	24/08/2006	148	41	11484	3538	3604	226	15314	8.6	11.8	0
13	23/01/2007	161	44	12322	3608	3536	381	16239	8.2	12.1	0*
14	26/06/2007	160	47	12366	2925	3697	282	16345	1.8	2.1	0
Total		1631	354	120967	36688	42111	5525	168603			5461

*: Six citywide cycles of vehicle-mounted ULV were also conducted.

During focal cycles 1–7, the presence of larvae and pupae was recorded on datasheets only, whereas in cycles 8–14 samples of larvae and pupae from each positive container were transported to the laboratory where they were identified to species after development to the adult stage. Immature stages in positive containers nearly always were *Ae. aegypti*. Additionally, starting on early 2006 (focal cycle 10), containers were classified into eight mutually exclusive categories: A, tires; B, tanks, barrels and drums; C, flower vases; D, construction materials and discarded vehicle parts; E, bottles, cans and plastic goods; F, wells; G, natural containers; and H, other types (mainly ceramic pots, tin cans, pieces of canvas, canvas pools and broken or unused appliances such as refrigerators or washing machines) so that the most productive container types could be identified. The focal treatment cycle 10 (started in mid-February 2006) was interrupted due to an emergency response to a region-wide dengue outbreak. Supplementary indoor and city-wide truck-based ULV spraying campaigns were carried out in March–May 2003, August–September 2004, March–May 2006, and February–May 2007; application dates and interventions specific details are summarized in Table 1 and Text S1.

#### Evaluation surveys

From 2003 to 2005 (cycles 1–7), selected teams among those conducting regular control operations assessed the impact of larviciding operations shortly after them (mean = 41.8 days; 95% confidence interval, 37.4–46.3) in a convenience sample of blocks distributed throughout the city (i.e., early post-intervention assessment surveys). The total number of houses inspected for early infestation was 5,933 and averaged 448 houses per cycle (SD, 411). This additional monitoring scheme did not include additional treatments and was subsequently phased out because it lengthened the duration of focal treatment cycles.

### Data analysis

The data collected over all cycles of focal treatment, monitoring and indoor house sprays were entered in an Access^R^ database to calculate the outcome parameters: the house index (i.e., the percentage of inspected houses that were positive for *Ae. aegypti* larvae or pupae); the Breteau index (i.e. the number of water-holding containers that were positive for *Ae. aegypti* larvae or pupae per 100 houses inspected); the percentage of all visited houses that were closed or vacant or whose occupants were unavailable on first visit; the percentage of all visited households who denied entry to premises, and of all visited houses that were treated with larvicides (temephos or BTI) in each cycle. The first and third quartiles of the citywide house and Breteau index were calculated with package Hmisc in R software [28]; quartiles were based on the appropriate larval index value at each neighborhood *j* weighted by the number of houses inspected at each *j* and focal cycle *i* (i.e., time post-intervention, range 1–14). Breteau and house indices were checked for normality and then transformed to logarithms to the base 10 of count+1. Two-tailed paired t tests were used to examine differences between larval indices recorded in the preliminary survey and the first focal treatment cycle at each neighborhood.

Odds ratios (OR) and incidence rate ratios (IRR) were estimated to assess the effect size of control interventions at each neighborhood *j* and cycle *i* compared with pre-intervention (baseline) values at each *j* and *i* = 1 by fitting random-effects regression models clustered by neighborhood to time-dependent larval indices using Stata 9.0 [29]. The use of random-effects models responds to the fact that households within a neighborhood roughly share the same environment and other undetermined characteristics that can create dependencies between responses (positive containers or houses) within the same geographic unit. The outcome measures were the Breteau and house indices at each *j* and cycle *i*. The main model included as explanatory variables the effects of focal cycle number (a categorical variable that compares each cycle 2–14 to the pre-intervention cycle 1); temperature and rainfall at different time lags before the exact onset of cycle *i* at *j* (see selection procedures below); the intensity of surveillance coverage at *j* and *i*−1 (i.e., a continuous variable measured by the proportion of total houses at *j* that were inspected at the immediately preceding focal cycle), and the corresponding baseline larval indices at *j* and *i* = 1 (pre-intervention). Pre-intervention larval indices were included as predictors based on the notion that the most infested neighborhoods before the intervention program may continue having more foci after interventions. The entire set of predictors was selected *a priori* based on existing knowledge. Variations in house and Breteau indices were tested with the commands glm (family binomial) and xtmixed using maximum likelihood procedures in Stata 9.1. The short-term effects of interventions on larval indices (as determined by the early post-intervention surveys at cycles 1–7) at fixed blocks within infested neighborhoods were assessed using the same procedures described before for each cycle separately. A dummy variable measured the effects of interventions at each cycle, and a time lag of 7 days was used for temperature and rainfall in all cycles.

Selection of the most adequate time lag and variables for representing weather effects was based on Akaike Information Criterion (AIC) [30]. Four sets of candidate variables were identified: mean daily temperature; minimum daily temperature; maximum daily temperature, and daily rainfall averaged for each neighborhood at every focal cycle. Each of these variables was computed for several weekly time lags (range, 0–7 weeks). The set of temperature variables was considered linearly or by adding a quadratic term to account for putative non-linear effects. For each set of weather-related variables, models including non-weather variables were fitted to Breteau indices for each time lag. The best lag was identified by the model with the smallest AIC. Models with every combination of the four selected lagged variables were computed in order to select the best model including all the variables considered.

## Results

Mean house (19.5%) and Breteau indices (22.5) at the preliminary survey in late 2002 were consistent with those recorded before interventions in late 2001 (22% and 54, respectively). Neighborhood-specific Breteau indices decreased between the preliminary survey and the first focal cycle before larviciding operations but the difference was not statistically significant (t = 1.59, df = 33, P = 0.122). In contrast, the observed decrease in neighborhood-specific house indices was significant (t = 2.42, df = 33, P = 0.021). Mosquito larvae or pupae collected during surveys were predominantly *Ae. aegypti*; only *Culex sp.* (but no *Aedes albopictus*) were detected in a few containers. House and Breteau indices recorded at the first focal cycle varied widely between neighborhoods (coefficient of variation, 70% and 89%, respectively). The distribution of Breteau indices was bimodal and highly skewed, with 10 neighborhoods exceeding 30.

Between late 2002 and 2007, ≈170,000 households were visited and of these, 120,000 were surveyed for *Ae. aegypti* and 37,000 were treated with larvicides (Table 1). Household inspections per cycle averaged 8,511 (SD, 2,127; range, 5,587 to 12,366) (Table 1 and Figure S2). On average, 25% (SD = 2%) of the houses visited in each cycle were closed or vacant or with householders temporarily absent on first visit, and 3% (SD = 1%) denied entry for inspection of premises (Figure S2). The denial rate declined from 7% at baseline to 3% at 21 months post-initial intervention (MPI) (focal cycle 6, November 2004) but increased again to 5% at 29–33 MPI (cycles 8–9, June–October 2005), only to decline again to 1–2% at 41–47 MPI (cycles 12–14, August 2006–June 2007) when a dengue outbreak emerged in Paraguay in early 2006. The percentage of visited houses that were treated with larvicides averaged 22.2% (SD = 2.8%). The ratio between larvicide-treated houses and *Ae. aegypti* positive-houses averaged 5.8 (SD = 3.1) but varied widely from 2.8 to 13.5 over time. The total number of positive containers detected (mean = 738, SD = 418) and the kilograms of temephos applied (mean = 193 kg, SD = 45) at each focal cycle were not significantly correlated (r = 0.350, n = 14, P>0.1).

**Figure 2:**
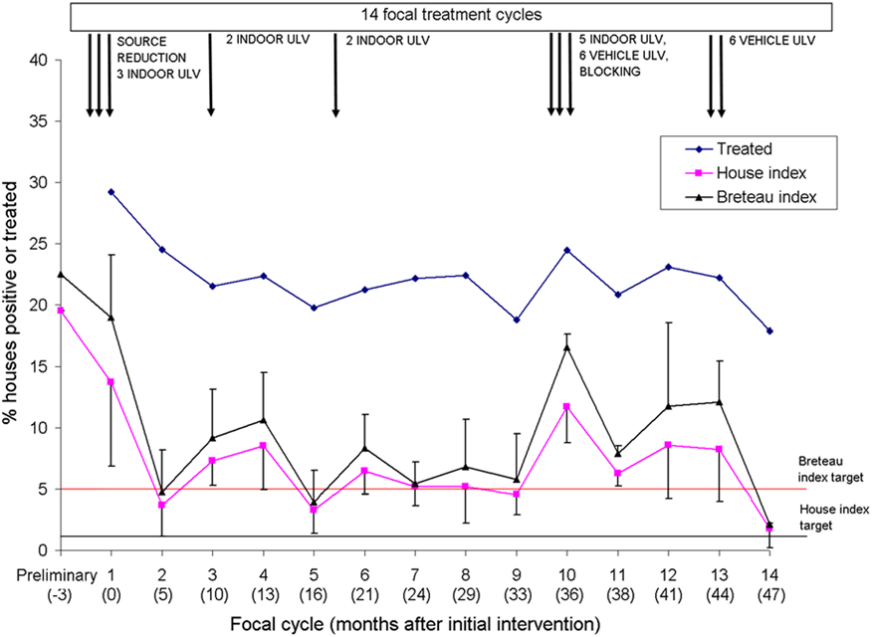
Distribution of mean house and Breteau indices, percent of houses treated with larvicides, and control interventions in Clorinda, Argentina, 2003–2007.

Citywide house indices declined sharply from 13.7% at baseline to 3.7% at the second focal cycle conducted mostly through spring of 2003 whereas Breteau indices declined from 19.0 to 4.8 (Figure 2). Larval indices then fluctuated seasonally and peaked every year between summer and early fall, with large variations between neighborhoods within anyone focal cycle as expressed by interquartile ranges. Monthly house and Breteau indices at a citywide scale over the five years were highly positively correlated (r = 0.966, n = 60, P<0.001).

Weather-related variables exerted highly significant effects on larval indices, especially when time lags were allowed for (Table 2). Based on AIC scores, the best lags were 1 week for mean daily rainfall and mean minimum daily temperature, and 4 weeks for mean daily temperature and mean maximum daily temperature. In every case the best lag identified clearly surpassed other candidate lags (range of ΔAIC of weather variables, 5.6–11.0). Because substantial model selection uncertainty was found when comparing models including temperatures with and without quadratic terms, the linear variables were selected for the remaining analyses. Using the selected time lags for each variable, the best model found included mean rainfall, mean temperature and mean minimum temperature. All of the selected variables had a positive association with larval indices (Table 3).

**Table 2:** Akaike Information Criterion scores of the models for Breteau indices using weather variables with different time lags.

Lag (weeks)	Mean rainfall	Mean temperature		Minimum temperature		Maximum temperature	
		Linear	Linear+quadratic	Linear	Linear+quadratic	Linear	Linear+quadratic
0	55.7	19.0	20.1	13.7	13.9	30.0	25.9
1	33.7	10.8	12.7	0.0	1.0	26.2	28.0
2	54.8	20.4	21.5	10.8	10.8	31.9	32.8
3	48.7	14.7	16.6	11.0	13.0	24.5	24.1
4	52.0	5.2	4.8	12.4	8.0	8.2	9.1
5	56.3	11.9	9.7	18.2	19.0	13.8	9.3
6	55.1	22.3	23.0	32.5	31.0	19.1	21.0
7	56.1	26.2	20.6	33.6	33.2	14.2	15.0

**Table 3:** Random-effects multiple regression models of the effects of focal treatment cycles on house indices (logistic model) and Breteau indices (linear model) relative to cycle 1 in Clorinda, Argentina, 2003–2007.

Explanatory variables	House index				Breteau index			
	OR	95% confidence interval		*P*-value	Coefficient	95% confidence interval		*P*-value
Mean temperature at lag 4	1.040	1.011	1.071	0.007	0.028	0.014	0.042	0.000
Mean minimum temperature at lag 1	1.054	1.036	1.072	0.000	0.035	0.020	0.050	0.000
Mean rainfall at lag 1	1.023	1.014	1.032	0.000	0.014	0.008	0.020	0.000
Treatment								
Focal 2	0.397	0.262	0.603	0.000	−1.132	−1.540	−0.724	0.000
Focal 3	0.544	0.358	0.828	0.005	−0.834	−1.222	−0.446	0.000
Focal 4	0.994	0.559	1.766	0.983	−0.482	−0.889	−0.074	0.020
Focal 5	0.467	0.255	0.857	0.014	−1.078	−1.483	−0.673	0.000
Focal 6	0.419	0.308	0.569	0.000	−0.905	−1.309	−0.501	0.000
Focal 7	0.269	0.183	0.397	0.000	−1.343	−1.740	−0.945	0.000
Focal 8	0.823	0.403	1.677	0.591	−0.811	−1.215	−0.406	0.000
Focal 9	0.345	0.201	0.592	0.000	−1.263	−1.661	−0.866	0.000
Focal 10	0.990	0.700	1.400	0.955	−0.012	−0.573	0.334	0.606
Focal 11	0.731	0.468	1.143	0.169	−0.354	−0.671	−0.037	0.029
Focal 12	0.793	0.461	1.365	0.403	−0.359	−0.757	0.039	0.077
Focal 13	0.640	0.334	1.228	0.180	−0.693	−1.115	−0.270	0.001
Focal 14	0.129	0.076	0.218	0.000	−1.882	−2.318	−1.445	0.000
Baseline larval index	1.008	0.983	1.033	0.553	0.374	0.254	0.494	0.000
Surveillance coverage	0.522	0.345	0.789	0.002	−0.141	−0.560	0.279	0.512

Control actions exerted significant impacts on larval indices, with exception of a limited upsurge at 36 MPI (cycle 10, February 2006) (Figure 2). When post-intervention larval indices at focal cycles 2–14 were compared to pre-intervention indices at cycle 1 by random-effects multiple regression, log-transformed Breteau indices declined significantly (P<0.05) or highly significantly (P<0.001) in all cycles except cycle 10 and 12 after allowing for lagged effects of temperature and rainfall (P<0.001), baseline Breteau index (P<0.001), and surveillance coverage (P = 0.512) at the preceding focal cycle (Wald χ^2^ test, df = 18, P<0.001) (Table 3). Significant heterogeneity between neighborhoods was indicated by the standard deviation of the μ parameter representing between-cluster variations (Likelihood ratio test, χ^2^ = 89.5, df = 1, P<0.001). The residual intraclass correlation (ρ) was 0.30, thus indicating that 30% of the variance in post-intervention log Breteau indices that is not explained by the covariates is due to time-invariant neighborhood-specific characteristics. House indices were significantly reduced by 13–54% relative to pre-intervention indices in cycles 2–3, 5–7, 9, and 14. Post-intervention and pre-intervention Breteau indices were positively and significantly associated, and post-intervention house indices were negatively and significantly associated with surveillance coverage at the preceding cycle.

**Figure 3:**
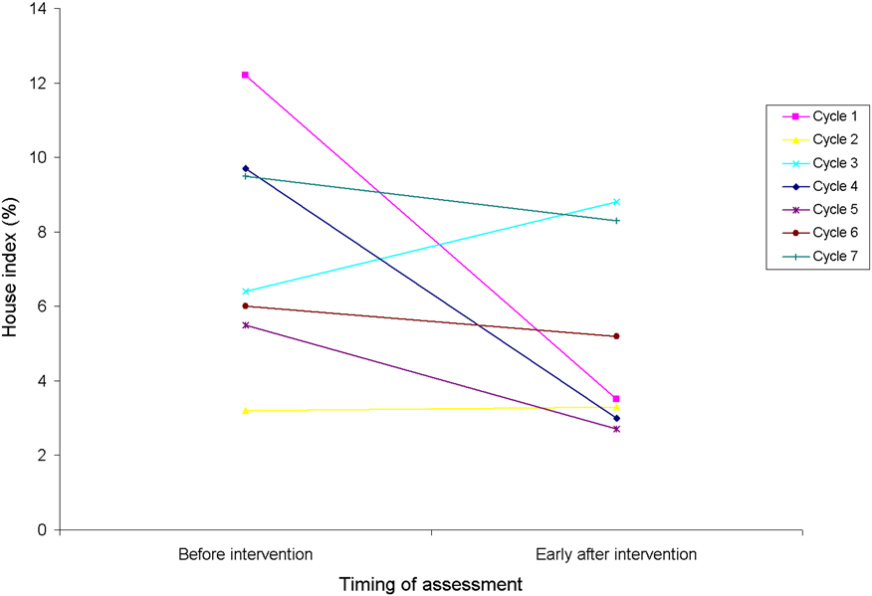
Short-term impact of larviciding and manual operations on larval indices at the same blocks in focal treatment cycles 1–7 in Clorinda, Argentina, March 2003–April 2005.

The early post-intervention surveys after focal cycles 1–7 showed that larval indices seldom fell to 0 at the same infested spatial units shortly after treatment (Figure 3). Random-effects multiple regression showed that pre-intervention house indices were reduced significantly at focal cycles 1 (from 12.2% to 3.5%, P = 0.001) and 4 (from 9.7% to 3.0%, P = 0.047), with borderline significant effects at cycle 5 (P = 0.057, from 5.5% to 2.7%). Average pre-intervention house indices were barely reduced at other cycles (P>0.3). Breteau indices showed similar patterns.

**Figure 4:**
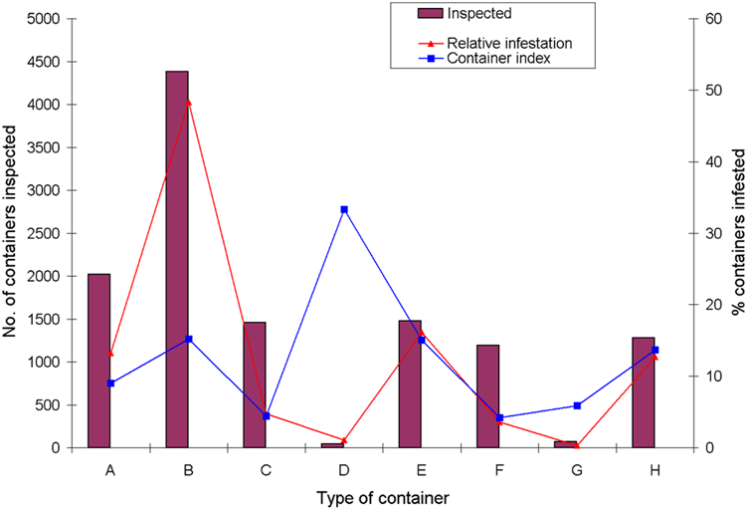
Distribution of the number of containers inspected and infested by *Ae. aegypti* according to type of container at focal treatment cycle 12 in Clorinda, Argentina, August 2006–January 2007.

The abundance and infestation of water-holding container types and distribution of larval indices differed largely among types of container. For example, at focal cycle 12 (spring–summer 2006–2007), tanks, barrels and drums for water storage (B type) were the most abundant containers (4,380) and the most likely to be infested (15.2%), accounting for 49% of all infested containers found (Figure 4). The second most important container class was disposable bottles, cans and plastics (E type), which were abundant (1,476) and as frequently infested (15.0%) as B containers, but accounted for only 16% of all infested containers. The frequency distribution of containers per type in cycle 12 was highly overdispersed between neighborhoods, with coefficient of variations that increased from 36% (type B) to 288% (D).

**Figure 5:**
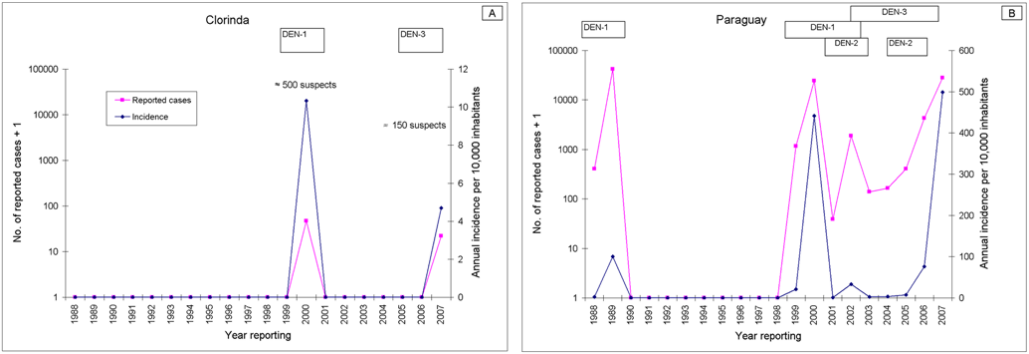
Reported cases of dengue and incidence of dengue in Clorinda, Argentina, and Paraguay, 1988–2007.

The reported incidence of classic DF cases in Clorinda declined from 10.4 per 10,000 inhabitants in 2000 (46 confirmed cases including 20 autochthonous cases by DEN-1, and about 500 suspect cases) to 0 from 2001 to 2006 [22],[27], and then increased up to 4.5 cases (by DEN-3) per 10,000 in January–April 2007 [31] (Figure 5A). Of 267 suspect DF cases in 2007, 21 were confirmed (including only 5 autochthonous cases); 86 were excluded as dengue, and 160 remained without confirmation [31]. Meanwhile in Paraguay, following the 1999 outbreak with 1,164 reported cases, 24,282 cases by DEN-1 were reported in 2000 (incidence, 49.3 per 10,000) though estimates ranged up to 300,000 [32] (Figure 5B). This outbreak was followed by a low-level transmission period with decreasing number of cases from 1,871 in 2002 (by DEN-1, DEN-2 and DEN-3), to 137 and 164 cases in 2003 and 2004 (by DEN-3), and another upward trend with 405 cases in 2005 (by DEN-2) and 4,271 cases (by DEN-3) in 2006 [11]. In Asunción, with high infestation levels, 1,700 DF cases were reported in 2006 [12], and the incidence of DF was 135.7 per 10,000 inhabitants in January–April 2007 [13]. The 2007 outbreak included 28,130 reported cases by DEN-3 (up to week 21, several with unusually severe manifestations), 55 confirmed cases of DHF/DSS, and 17 deaths probably related to dengue [33].

## Discussion

The citywide intervention program in Clorinda (i) exerted a significant impact on larval indices with respect to pre-intervention levels but failed to keep them below the desired target levels (especially during summer in some neighborhoods) despite the extended use of temephos and additional actions; (ii) achieved sustained community acceptance; (iii) most likely averted new dengue outbreaks between 2003 and 2006, and (iv) limited to a large extent the 2007 outbreak of DEN-3 in an immunologically naive population. Failure to reach the desired target levels occurred in the absence of significant insecticide resistance in *Ae. aegypti* populations from Clorinda, where the resistance ratios to temephos steadily increased from 1.31 in 2004 to 3.10 in 2007 [34].

The intervention program exerted a greater, more sustained impact than the time-limited vector control campaigns conducted in late 2001, whose effects were short-lived and undetectable by late 2002. Post-intervention larval indices during every late summer from 2001 to 2005 were much lower in Clorinda than in Tartagal and Orán (Salta), where in spite of larviciding efforts mean house and Breteau indices peaked at 27–35% and 20–130, respectively [35]. The seasonal dynamics of infestation documented in Clorinda, with peaks in late summer is overall consistent with data recorded in Salta [35],[36] and Iguazú (H. D. Coto, unpublished data). This appears to be a generalized regional pattern despite local differences in the main types of infested containers.

The putative reasons for failing to keep larval indices below target levels in Clorinda are multiple:

1. Incomplete surveillance coverage. Vector surveillance and insecticide treatment coverage were high but incomplete in space and time despite the community's high levels of acceptance of suppressive measures. House indices were inversely related to surveillance coverage. Houses lost-to-inspection may have constituted a persistent source of infestations of unknown magnitude. Re-visiting houses at appropriately scheduled times increased surveillance coverage from late 2006 forward. Despite its importance elsewhere [37], intensified searches for alternative developmental sites in non-residential habitats (tree holes and bromeliads) yielded negative results, and only 3.4% of indoor containers were found to be infested in early fall 2007 (unpublished results);
2. Limited residuality of temephos. The expected duration of temephos residual effects (about three months) and standard control guidelines, further compounded with operational restrictions, dictated that each focal treatment cycle took an average of almost four months. However, the residuality of temephos has recently been shown to vary widely between <1 and 6 months depending on the type of formulation and container [38]–[40], and is much more limited in the field [41]. In Clorinda, the occurrence of infested containers mostly within 46 days after larviciding operations at the same blocks during focal cycles 1–7, combined with preliminary results of bioassays in treated tanks (Garelli, unpublished) , suggest that the actual residuality of temephos is much shorter and more variable than assumed. The fast turnover rate of potable water stored in tanks during spring and summer may have also contributed to reduced residual effects;
3. Permanent developmental sites. In the absence of major changes in the management of large containers for permanent water storage, these mosquito developmental sites were continuously available, rainfall-independent, and varied little in frequency among neighborhoods. Unfortunately, the intervention program did not seek to identify the most productive containers for targeted control, and the intervention protocol only suffered minor adjustments over time. Pupal surveys conducted in a large neighborhood in 2007 showed that large tanks used for potable water storage were both the most abundant and the most productive type of container [42];
4. Suitable climatic conditions. The prevailing local weather conditions are very favorable for *Ae. aegypti* (as shown by the presence of immature stages on every month over the five years), and would determine fast egg-to-adult developmental times ranging from nearly 6 days in summer up to 10 days in early spring or fall according to experimental data [43]. The gonotrophic cycle length of female *Ae. aegypti* would have been 3.8 days at local mean temperatures (27°C) over January–March 2003–2007, as interpolated from tables in [44]. Larval indices were highly significantly associated with average minimum temperatures and rainfall one week earlier and average temperatures 4 weeks earlier. These data suggest that recent cool temperatures and rainfall influenced variations in *Ae. aegypti* larval indices, as other studies also showed for female and larval abundance [45],[46]. Fast development rates and the limited residuality of temephos during hot weather jointly create a window of opportunity in which the vector population recovers partially between focal cycles and then spreads by flight dispersal at a local scale [47];
5. Limited source reduction efforts. These efforts occurred mainly at the outset of the intervention program. Householders' response to cleanup messages and removal of containers was neither assessed nor coupled with improved solid waste disposal by municipal authorities, which facilitated the persistence of potential developmental sites;
6. Lack of regular perifocal residual spraying with insecticides. Only indoor ULV spraying with partial or minimal coverage was conducted sporadically and therefore probably exerted negligible effects on subsequent larval indices;
7. Lack of adequate, sustained community participation beyond mere acceptance of regular control measures albeit at high levels.

Larval indices decreased more sharply both in relative and absolute terms immediately after the multiple, high-coverage control actions executed at focal cycle 1 than at subsequent cycles focusing mainly on larviciding operations and eventually adding ULV sprays. The actual effectiveness of vehicle-mounted ULV sprays has been questioned repeatedly [2],[8],[47] and was not specifically assessed in Clorinda. However, *a posteriori* comparisons of Breteau indices (Table 1) between successive focal cycles conducted during summer-fall (cycles 1–2, 4–5, 7–8, 10–11 and 13–14) show maximum percent relative reductions between cycles 13–14 (83%, including high larviciding coverage and six citywide cycles of vehicle-mounted ULV sprays) and cycles 1–2 (75%; intense source reduction and incomplete indoor ULV), followed by cycles 4–5 (63%, no indoor ULV) and 10–11 (52%, partial larviciding, blocking, marginal indoor ULV and six citywide cycles of vehicle-mounted ULV sprays), whereas cycles 7–8 showed a 24% relative increase (marginal indoor ULV). In conclusion, maximum impact was achieved through multiple, high-coverage control actions including surveillance and larviciding efforts combined with either intense source reduction or repeated citywide cycles of vehicle-mounted ULV sprays.

The upsurge in larval indices at focal cycle 10 reflects the limitations of chemical control and the well-known capacity of population recovery of *Ae. aegypti*. This upsurge is probably explained by a relative decrease in surveillance and larvicide treatment coverage over the two previous focal cycles 8 and 9 combined with a net increase in rainfall of 106 mm between December 2005 and March 2006, at very similar period temperatures as those recorded over 2003–2005. In contrast, the increasing surveillance and larvicide coverage achieved over focal cycles 11–13 combined with repeated vehicle-mounted ULV sprays at cycle 13 and more sustained cool temperatures in the driest winter 2007 preceded the lowest larval indices ever recorded during the five-year period (1.8% and 2.1). Therefore, several factors likely contributed to the sharp decrease in larval indices at focal cycle 14.

Infestations varied largely between neighborhoods throughout the intervention period, with a few neighborhoods having persistently high indices over time. Such heterogeneities were uncovered by the random-effects multiple regression model. More peripheral neighborhoods with more discontinuous water service tended to display larger and more variable larval indices. An unreliable, discontinuous water service has been recognized as a major determinant of persistent developmental sites in Venezuela [48]. Other environmental and socio-demographic determinants may have also contributed to the complex infestation dynamics observed. Heterogeneities in infestation and dengue transmission risk have been uncovered elsewhere [49],[50] and may lead to developing more cost-effective, targeted control programs.

House and Breteau indices gave a consistent picture of infestation levels over time, and in 2007 were positively correlated with pupal counts at the block level, though not very strongly so [42]. Although the Breteau index was once considered the most informative measure for monitoring control operations [7],[51],[52], both indices have limitations when used to assess the quantitative impact of control actions, partly because they are based on presence-absence of immature stages [53]. Although it is often difficult to show significant intervention effects on larval indices, the post-intervention reductions observed in most focal cycles were substantive and statistically significant.

Human infection usually is the crucial outcome for evaluating the impact of vector suppressive actions. The sustained intervention program most likely averted the occurrence of major dengue outbreaks in Clorinda from 2003 to 2006 despite of the vicinity of endemic dengue transmission in Paraguay during the same time period; the large daily movement of people across the border; the invasion of the new DEN-3 serotype into Brazil, Bolivia and Paraguay since 2001–2002, and the very low herd immunity to DEN-1 and DEN-3 in the Clorinda population. When a major DEN-3 outbreak emerged in Paraguay in early 2006, the ongoing larval suppressive actions supplemented by limited source reduction and ULV space spraying averted a local outbreak and also limited the apparent number of DF cases in 2007. These results need to be interpreted with caution and may constitute a reasonable circumstantial case, considering that the apparent incidence of DF in 2007 was both much lower than in 2000 and than in the concurrent epidemic in Asunción. Moreover, most of the 21 confirmed DF cases in Clorinda in 2007 reported recent travel history to Paraguay and were not considered autochthonous cases. In addition, the apparently random spatial distribution of autochthonous or suspect cases throughout the city [31] argues against intense local viral transmission. Vector control efforts apparently reduced the likelihood of localized outbreaks initiated from individuals who became infected in Paraguay, but Clorinda is rather small and this by itself could have reduced the likelihood of dengue transmission. Whether the several layers of heterogeneity detected in Clorinda reduced the likelihood of an epidemic remains an open question.

Dengue transmission risks may be measured by entomologic biting rates [44],[52]. At mean ambient temperatures (27°C) in Clorinda during January–March 2003–2007, the expected extrinsic latent period of the virus in the mosquito would be 12.9 days, and the estimated entomologic threshold for dengue transmission (assuming 0% of dengue antibody prevalence) would only range from 0.7 to 0.83 *Ae. aegypti* pupae per person in January–May 2007 (interpolated from tables in [44]). A house index <1% and a Breteau index <5 were suggested as dengue transmission thresholds [9],[54], and a maximum Breteau index ≥4 at the block level was considered a suitable predictor of dengue transmission in Cuba [55]. If these transmission thresholds held in our study area, several sections of most neighborhoods would have been well above threshold during every summer, when cases typically peak in the region [33],[56]. However, dengue transmission thresholds are potentially modified by several factors. Some of the implicated parameters are hard to measure in the field, and its estimates are affected by large sampling errors and other sources of bias acting multiplicatively (i.e., increased uncertainty around threshold estimates) [57]. Entomologic transmission thresholds need further validation [44],[52] at various spatial scales before they are used operationally.

Some problems limit the interpretation of our results. Quantitative data on manual treatment of containers and container-based coverage were not collected. The study design is a before-and-after community intervention with no internal control arm; the latter was not justified in view of the impending risk of another dengue outbreak in Clorinda. In practice, neighboring Paraguay served as a surrogate external control area for comparison of reported incidence rates given the limited vector suppressive actions conducted there. Before-and-after comparisons are potentially biased by temporal trends in vector density [58] but our multivariate analysis of intervention effects on larval indices accounted for weather effects. The reported incidence of dengue in Clorinda in 2007 was much lower than during the first local outbreak in 2000, but exactly how much is uncertain because no cohort study was conducted to establish the actual incidence of infection. Intense movement of people across the border with Paraguay seriously undermines the capacity to establish whether an infection was autochthonous or imported. The assessment of intervention effects may be further complicated by underreporting of asymptomatic or mild dengue infections, imported infections, and misclassification of other febrile illnesses with overlapping clinical features, such as influenza. The net effects of different sources of bias running in opposite directions are hard to assess. Febrile cases unrelated to dengue were 2–3 times as frequent as DF cases during the 2000 summer outbreak in Clorinda [27] and in 2007. Silent transmission of dengue viruses is more likely in populations without previous dengue, especially if caused by strains with low virulence [3]. However, enhanced community awareness and continuous passive surveillance of febrile syndromes after the 2000 outbreak suggest that the absence of reported or suspect DF cases during 2003–2006 may at most represent marginal occurrence of local transmission over such period.

A major feature of the five-year intervention program is that it was mostly supported and run citywide in a high-risk area by an external organization in close cooperation with the local Municipality and the Ministry of Health. The program was thus an example of highly desirable intersectoral cooperation [59]. It was initiated and consolidated in the context of a severe socio-economic crisis in a border area, and succeeded in maintaining high levels of community acceptance over time. The latter in part may be attributed to the education and promotion campaign launched at the outset of operations, further enhanced by community awareness of the threat of a dengue outbreak spilling over from Paraguay. The fact that most of the vector control personnel were women from the same community may account for the very low mean fraction of households that denied entry to premises (3%) even in neighborhoods whose residents frequently did not allow the labor of municipal agents inside house premises. House-to-house larval surveys are typically plagued by difficulties of access. Issues of acceptability, coverage and delivery, which frequently compromise the effectiveness of the available vector control tools, were minimized in the Clorinda experience. However, the effective surveillance coverage of closed or vacant houses remains to be addressed. The intervention program trained a significant number of local human resources for future transference of control routines to official health services. Gaps in this transference or interruption of vector control actions are predicted to pave the way to rapid recovery of *Ae. aegypti* populations and eventual local dengue outbreaks.

### Implications for dengue vector control and research

Gubler [2] noted that “… The sporadic nature of dengue epidemics and the misguided reliance on using insecticidal space sprays to kill adult mosquitoes prevented most countries from developing and implementing programs that focused on larval mosquito control, which were much more difficult to implement and maintain”. Here we advocate that such programs are still needed; they should be run permanently with high coverage, especially in a hyperendemic context with regional expansion of DHF/DSS cases, and may be mostly maintained with locally available resources, duly coordinated and supervised. This does not preempt the fact that governments must invest much more resources and efforts on scientifically-based vector control programs run by qualified personnel than they have done so far. Although the Clorinda intervention program did not reach target levels, it had a positive impact on public health because it prevented the serious dengue outbreaks that occurred in neighboring countries during the study period. Without the interventions, at the reported incidence rates in Paraguay the situation in Clorinda would have been much worse.

The Clorinda experience was successful at some program levels and left some lessons for further improvement. The use of controlled-release insecticide formulations or sachets that are retrievable during cleaning and washing of water-storage containers would extend the residual activity of temephos [40]. There is a great need of new, more effective larviciding products that last longer, but they will also face water management and coverage issues. The upward trend observed in temephos resistance indicates that resistance management schemes should be developed and considered in the near future, because RRs around 3 do not revert spontaneously to pre-intervention susceptibility levels. Resistance to temephos with potential cross-resistance to other insecticides has expanded greatly and caused repeated control failures in Brazil [60],[61], and has been detected elsewhere in Argentina [62]. Pyriproxyphen is a relevant candidate for larvicide replacement [10]. Identification of neighborhoods at increased risk of infestation and transmission are needed for developing more cost-effective, targeted control strategies. Several sources of heterogeneity pose major challenges to the control of *Ae. aegypti*. The implemented program was born as a community-based intervention by some definitions [58] and gradually turned into a top-down vector control program suffering from excessive reliance on insecticides. In Clorinda, current interventions should evolve towards a multifaceted integrated program with intensified coverage, source reduction and environmental management measures, such as providing lids or insecticide-treated covers to water storage containers [10],[63]. Such integrated program needs also strengthened communication and health education components [64] and a broad social participation aiming at long-term sustainability. Other strategic solutions include development of the infrastructure for providing potable water and improved disposal of solid waste.

Vector control and disease management must remain a regional effort to prevent “spillovers” such as those from Asunción to Clorinda or from Brazil to Paraguay, within the frame of sustainable development rather than being viewed exclusively as a matter of health [59]. These issues are common to other neglected tropical diseases affecting vulnerable populations in the Gran Chaco region over Argentina, Bolivia and Paraguay [65]. The price of not establishing regional control efforts of a more permanent nature has lead to the predicted [3] and observed expansion of DHF/DSS in the Americas, which reached Paraguay in 2007.

## Supporting Information

Alternative Language Abstract S1 Translation of the Abstract into Spanish by Ricardo E. Gürtler.
(0.02 MB DOC)

Figure S1 Map of Clorinda, Formosa, Argentina, its neighborhoods and location relative to Asunción, Paraguay.
(5.62 MB TIF)

Figure S2 Percentage of houses inspected for larval infestations, closed or vacant, and refusing access for inspection at focal treatment cycles 1–14 in Clorinda, Argentina, 2003–2007.
(0.26 MB TIF)

Text S1 Supplementary methods.
(0.03 MB DOC)
