# Supplementary material for: Effects of a Five-Year Citywide Intervention Program To Control Aedes aegypti and Prevent Dengue Outbreaks in Northern Argentina
Source: PLoS Negl Trop Dis. 2009 Apr 28;3(4):e427. doi: 10.1371/journal.pntd.0000427 (PMC2669131; doi:10.1371/journal.pntd.0000427)
Supplement: Alternative Language Abstract S1 — Translation of the Abstract into Spanish by Ricardo E. Gürtler. (0.02 MB DOC) [file pntd.0000427.s001.doc]

"Translation of the abstract into Spanish by RE Gürtler" and listed at the end of the

manuscript together with any other Supporting Information files.

**Resumen**

***Antecedentes:*** El dengue se ha propagado ampliamente a través de las Américas. La mayoría de los países no ha sido capaz de mantener programas de control de larvas de mosquitos en forma permanente, y los efectos a largo plazo de las acciones de control raramente han sido documentados.

***Metodología:*** El diseño del estudio se basó en una evaluación antes-después de los índices larvales de *Aedes aegypti* y de la incidencia notificada de dengue en toda la ciudad de Clorinda, noreste de Argentina, durante 2003-2007. Las intervenciones se basaron principalmente en el tratamiento focal con larvicidas de todos los sitios de cría de los mosquitos cada cuatro meses (14 ciclos), combinado con esfuerzos limitados de reducción de fuentes y aplicación de insecticidas a ultra bajo volumen durante operaciones de emergencia. El programa realizó en las viviendas 120.000 búsquedas de sitios de cría de mosquitos y 37.000 aplicaciones de larvicidas.

***Principales Hallazgos:*** Modelos de regresión con efectos aleatorios mostraron que los índices de Breteau disminuyeron significativamente en casi todos los ciclos focales respecto a los índices pre-intervención agrupados por vecindario, luego de tomar en cuenta los efectos retrasados de la temperatura y la precipitación, los índices de Breteau pre-intervención, y la cobertura de la vigilancia. Se halló una significativa heterogeneidad entre vecindarios. Los índices larvales raramente cayeron a 0 poco después de las intervenciones en las mismas manzanas. Los recipientes grandes para almacenar agua fueron los más abundantes y los que se hallaron infestados con mayor probabilidad. La incidencia de casos de dengue notificada se redujo desde 10,4 por 10,000 en 2000 (por DEN-1) a 0 entre 2001 y 2006, y luego aumentó a 4,5 casos por 10.000 en 2007 (por DEN-3). En el vecino Paraguay, la incidencia de dengue notificada en 2007 fue 30,6 veces mayor que en Clorinda.

***Conclusiones:*** Las intervenciones de control ejercieron un impacto significativo sobre los índices larvales pero no lograron mantenerlos por debajo de los niveles deseados durante cada verano; lograron una sostenida aceptación de la comunidad; muy probablemente previnieron nuevos brotes de dengue entre 2003-2006, y limitaron en un alto grado el brote de 2007. Para nuevos progresos, es necesario un cambio hacia un programa multifacético que incluya una intensificación de la cobertura y reducción de fuentes; el uso de tapas o coberturas tratadas con insecticidas en los recipientes usados para almacenar agua, y una amplia participación social que apunte a la sostenibilidad del programa a largo plazo.
