# Supplementary figures and images for: Effects of a Five-Year Citywide Intervention Program To Control Aedes aegypti and Prevent Dengue Outbreaks in Northern Argentina
Source: PLoS Negl Trop Dis. 2009 Apr 28;3(4):e427. doi: 10.1371/journal.pntd.0000427 (PMC2669131; doi:10.1371/journal.pntd.0000427)

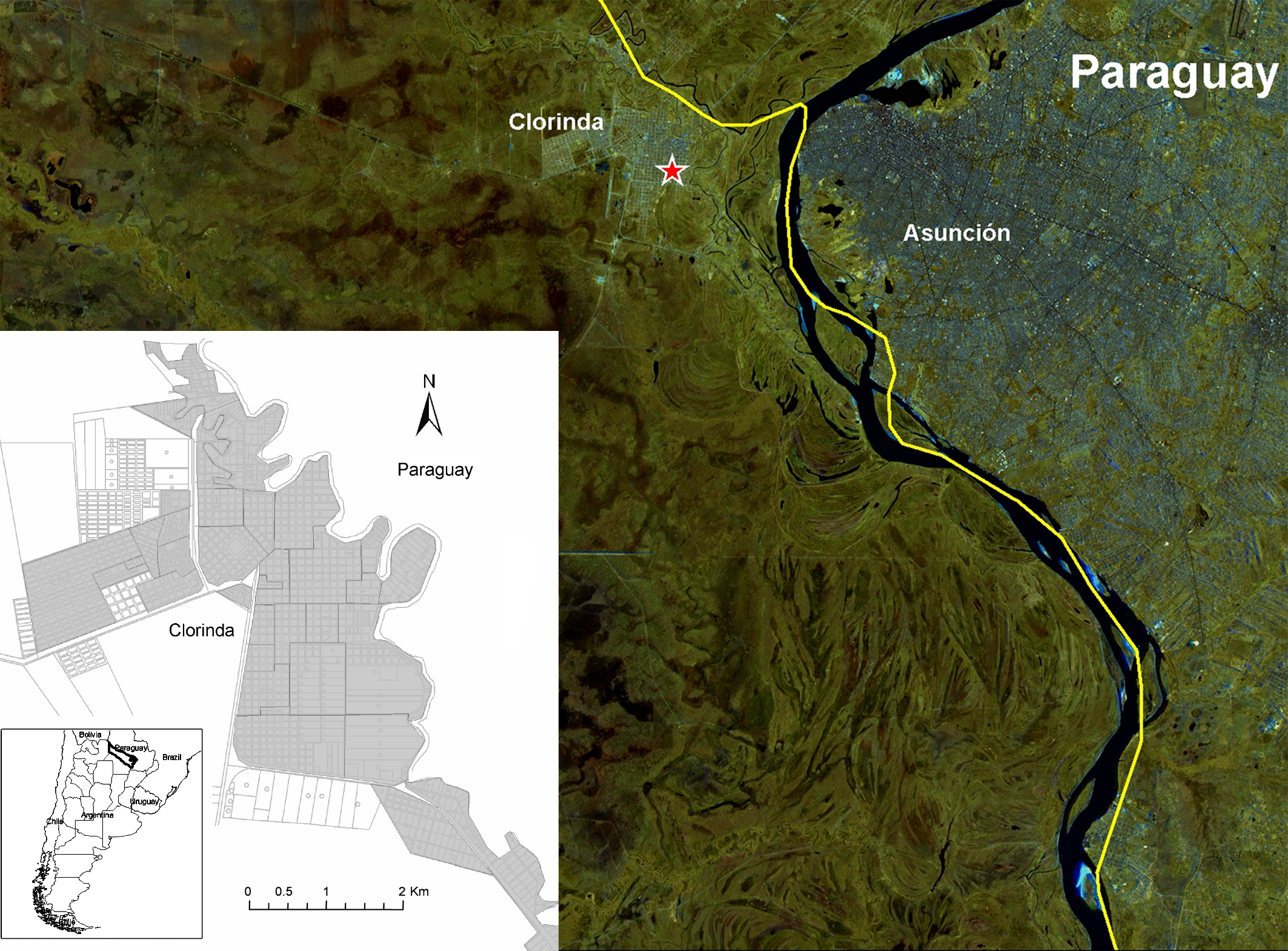

Supplement: Figure S1 — Map of Clorinda, Formosa, Argentina, its neighborhoods and location relative to Asunción, Paraguay. (5.62 MB TIF) [file pntd.0000427.s002.tif]

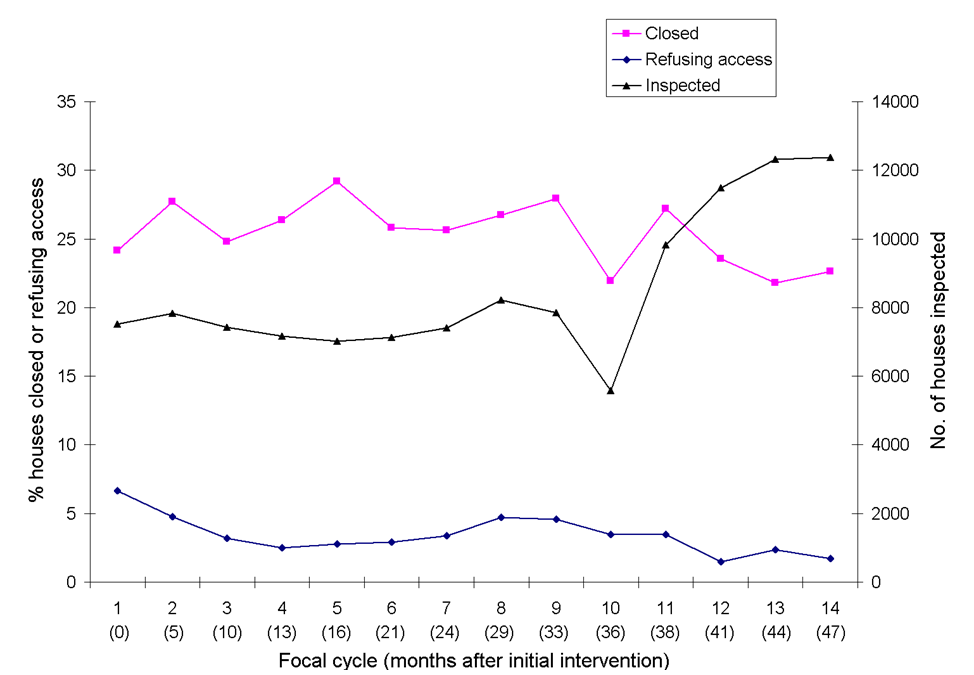

Supplement: Figure S2 — Percentage of houses inspected for larval infestations, closed or vacant, and refusing access for inspection at focal treatment cycles 1–14 in Clorinda, Argentina, 2003–2007. (0.26 MB TIF) [file pntd.0000427.s003.tif]
