# Supplementary material for: Effects of a Five-Year Citywide Intervention Program To Control Aedes aegypti and Prevent Dengue Outbreaks in Northern Argentina
Source: PLoS Negl Trop Dis. 2009 Apr 28;3(4):e427. doi: 10.1371/journal.pntd.0000427 (PMC2669131; doi:10.1371/journal.pntd.0000427)
Supplement: Text S1 — Supplementary methods. (0.03 MB DOC) [file pntd.0000427.s004.doc]

Supplementary methods

Other Interventions. The occurrence of suspect DF cases in Paraguay and high larval indices during late summer 2003 prompted the initiation of indoor and perifocal ULV spraying with permethrin (Depe®, Chemotecnica, Spegazzini; 10% emulsionable concentrate) by specially trained local men supported by the same welfare program. The insecticide was mixed with water, and polietilenglicol 100 (6%) was added as antievaporant. Applications were carried with ULV spraying machines (Fontan Portastar, Motan) during five seconds per room between mid-March and end of May 2003 (Table 1). Insecticide spraying was typically welcomed by householders because it reduced other nuisance pests such as *Culex* mosquitoes. Supplementary indoor ULV spraying with insecticides was conducted with variable coverage at various focal cycles (Table 1), and intended to treat neighborhoods just covered by the focal cycle though at a slower pace. An additional cycle of focal treatment and removal of discarded containers in private and public premises was conducted in the largest neighborhood (Barrio Primero de Mayo) in August-September 2004 because it had shown persistently high larval indices. Facing massive dengue outbreaks in neighboring Paraguay, Bolivia and Brazil in early 2006, the Ministry of Health of Argentina in cooperation with provincial health services launched emergency operations in high-risk areas in northern Argentina including Clorinda. Intensified removal of discarded containers was promoted by municipal authorities and community leaders, with apparently marginal effects. Surveillance of febrile syndromes compatible with dengue continued at the local public hospitals. Indoor ULV space spraying with insecticides was conducted in the residences of suspect cases and in nine blocks around them (‘blocking’) in April-May 2006. Six citywide cycles of vehicle-mounted ULV space spraying with permethrin emulsionable concentrate (Imperator) were each conducted in March-April 2006 and in February-May 2007 (Table 1).
